# Ethnic Background and Overweight among 5-Year-Old Children: The “Be Active, Eat Right” Study

**DOI:** 10.1155/2013/861246

**Published:** 2013-10-10

**Authors:** Lydian Veldhuis, Mara van Dooremaal, Willemieke Kroeze, Carry M. Renders, Remy A. HiraSing, Hein Raat

**Affiliations:** ^1^Department of Public Health, Erasmus MC, University Medical Center Rotterdam, Dr. Molewaterplein 50, P.O. Box 2040, 3000 CA Rotterdam, The Netherlands; ^2^Institute of Health Sciences, Faculty of Earth and Life Sciences, VU University Amsterdam, De Boelelaan 1085, 1081 HV Amsterdam, The Netherlands; ^3^Department of Public and Occupational Health, EMGO Institute for Health and Care Research, VU University Medical Center, P.O. Box 7057, 1007 MB Amsterdam, The Netherlands

## Abstract

*Introduction*. This study investigates the association between ethnic background and overweight (obesity included) among 5 year olds. *Methods*. We used baseline data from 5 year olds (*n* = 7801) and their parents collected for the “Be active, eat right” study. A child was considered to be of non-Dutch ethnic background when at least one of the parents was born abroad. Odds ratios (ORs) were adjusted for sociodemographic characteristics. *Results*. Compared to children of Dutch ethnic background, for children with a Moroccan ethnic background the OR for being overweight (obesity included) was 2.27 (95% CI 1.48–3.47), for Turkish children the OR was 3.63 (95% confidence interval (CI) 2.46–5.35), for Antillean children the OR was 1.97 (95% CI 1.01–3.86), and for Surinamese children the OR was 0.47 (95% CI 0.20–1.06). Addition of parental overweight decreased the ORs for Moroccan and Turkish children by 10.2% and 12.5%, and addition of watching TV and having breakfast by the child decreased the ORs by 7.9% and 12.2%. *Conclusion*. Already at a young age, children of Moroccan and Turkish ethnic background are at increased risk for being overweight compared to Dutch children. Parental overweight, watching TV, and not having breakfast by the child are contributing factors in this association.

## 1. Introduction

The prevalence of overweight among children is substantial in most parts of the world [1–3]. Overweight during childhood is associated with risk factors for cardiovascular disease, type 2 diabetes, psychosocial problems, impaired quality of life, being overweight as an adult, and high economic costs [2, 4]. In addition to behavioral, environmental, and socioeconomic risk factors [2, 5–8], being a child with an ethnic background different than the main ethnic group within a country may be a specific risk factor [9–14]. Among children in European countries, there is a large diversity of ethnic groups. Ethnic subgroups are often minority groups with a lower socioeconomic position than the main ethnic group within a country [9]. Lower socioeconomic position and minority status are suggested to be associated with a lower health status, including overweight [2, 8, 9, 15].

Recent studies on prevalence of overweight between subgroups of children of different ethnic background were conducted in non-European countries, mainly in the USA [11, 13, 16–20]. The studies from the US showed that, overall, Mexican American children and non-Hispanic black children are at increased risk for being overweight or obese, and that Asian American children have lower prevalences, compared to non-Hispanic white children. In Europe, there are differences between countries in the variety of nonnative ethnic subgroups and being overweight or obese. In the Netherlands, main non-Dutch subgroups are Moroccan, Turkish, Surinamese, and Dutch Antillean, and it has been reported that children from Moroccan and Turkish ethnic background are at increased risk for being overweight and obese [10]. Turkish is also one of the main ethnic subgroups in Germany with an increased risk for overweight [12], while in Austria no difference in risk between Austrian and Turkish children was found [14]. In the UK, Chinese children have a lower risk for obesity, while there is no consensus on whether South Asian or black children are at increased risk, compared to Caucasian children [21]. These European studies included mainly school-aged children [9, 14, 21]; research in children below 6 years is limited [10, 22, 23].

The determinants of ethnic disparities in childhood overweight and obesity remain poorly understood [19]. The prevalence differences between subgroups of different ethnic background are likely to be explained by characteristics of the children and parents related to material circumstances and behavior, which influence children's energy balance [11]. Parental weight status may be important in the association between children's ethnic background and weight status, as it represents shared genes and lifestyle [2]. The behaviors playing outside, watching TV, having breakfast, and drinking sweet beverages by the children have been shown to be associated with childhood overweight [24–28]. The study of the interplay between ethnic background and lifestyle-related behaviors of the children may be helpful for the development of effective prevention programs [6, 11, 17]. Only few studies reported to what extent parental overweight or lifestyle-related behaviors of the child contribute to the association between ethnic background and childhood overweight [12, 13, 16, 18, 23, 29, 30].

More research is needed to establish whether ethnic disparities in the prevalence of overweight are already present during early childhood in Europe, also with regard to a timely start of overweight prevention programs. Also research is needed to understand the underlying causes of ethnic disparities in overweight prevalence. Therefore the aim of our study was to investigate the association between ethnic background and overweight in a large sample of 5-year-old children in the Netherlands. Also investigated is the extent to which a potential association can be explained by parental overweight and lifestyle-related behaviors of the child.

## 2. Methods

### 2.1. Design and Study Population

This study is embedded in the “Be active, eat right” study. As detailed elsewhere [31], the “Be active, eat right” study aims to assess the effects of an overweight prevention program among children at elementary school throughout the Netherlands. The Medical Ethics Committee of the Erasmus MC (University Medical Centre Rotterdam, the Netherlands) approved the study protocol.

Of the 37 municipal health services in the Netherlands, nine municipal health services agreed to participate in the study. A total of 13,638 parents of 5 year olds were invited by mail for a free of charge well-child visit (attendance rate 95%) [32] at one of these nine participating municipal health services, and 64.4% (*n* = 8784) provided written informed consent to participate in the study. Baseline data of the children and their parents were collected during the 2007-2008 school year, and these data were used for the present study. Of the parents, 8683 (98.9%) completed a questionnaire with items on demographic, socioeconomic, and lifestyle-related characteristics of themselves and their child. Data on height and weight, measured by healthcare professionals during the well-child visit, was available for 8750 (99.6%) children. 

Of the in total 8784 children participating in the study, 141 children were excluded from the analysis because of missing data on height, weight, age, and/or gender, as this information was needed to determine the child's weight status (see below). Children were also excluded from analysis when data was missing on their ethnic background (*n* = 78). Children with a Moroccan, Turkish, Surinamese, or Dutch Antillean ethnic background were the largest non-Dutch ethnic subgroups (see below). Children with an “other Western” (*n* = 452) and an “other non-Western” ethnic background (*n* = 312) were excluded, because of the mixed composition of these groups. Finally, 7801 children were available for analysis. 

### 2.2. Ethnic Background of the Child

A child was considered to be of non-Dutch ethnic background when at least one of the parents was born abroad, as defined by Statistics Netherlands [33]. If at least one parent was born abroad and the child was also born abroad, the country of birth of the child determined the subgroup. If one of the parents was born abroad and the child was not, the country of birth of that parent determined the ethnic background. If both parents were born abroad and the child was not, the country of birth of the mother determined the ethnic background of the child. 

### 2.3. Weight Status of the Child

Body weight and height were measured by trained healthcare professionals of the municipal health services using standardized methods as described in a protocol [34]. Body weight was measured to the nearest 0.1 kilograms and height to the nearest 0.1 centimeter. Body mass index (BMI) was calculated by dividing weight (in kilograms) by height (in meters) squared. The weight status of the children was assessed according to the age-specific and gender-specific cutoff points for body mass index (BMI) as published by the International Obesity Task Force (IOTF) [35]. When a child's BMI value was the same as or higher than the lower-bound cutoff point for overweight for the child's age and gender, the child was classified as overweight (obesity included). Overweight and obesity were combined, allowing meaningful comparisons between the subgroups of different ethnic backgrounds.

### 2.4. Sociodemographic Characteristics, Parental Overweight, and Lifestyle-Related Behaviors of the Child

Sociodemographic characteristics of the child, parent, and family and parental overweight were considered potential confounders, and lifestyle-related behaviors of the child were considered potential mediators in the ethnic background-overweight association [1, 2, 5, 7, 8, 36–39]. Information on the gender and age of the child and the parent who completed the questionnaire, educational level, and height and weight of the parent and family situation were obtained by the questionnaire. Educational level of the parent was recoded in three categories according to the Dutch standard classification as defined by Statistics Netherlands [40]: low level (no education, primary school, lower vocational school, or intermediate general secondary school); midlevel (higher general secondary school or intermediate vocational training); and high level (higher vocational training or academic education). Self-reported height and weight of the parent were used to calculate BMI. Parents were classified as overweight (obesity included) when the BMI value was ≥25 kg/(m)^2^, as defined by the World Health Organization [3]. Family situation was recoded as a two-parent family or otherwise. 

Four lifestyle-related behaviors of the child were assessed also by the questionnaire completed by the parent. Parents reported the following behaviors of the child: playing outside (recoded as ≥1 hour/day, <1 hour/day), watching television (TV) (recoded as ≤2 hours/day, >2 hours/day), having breakfast (recoded as 7 days/week, <7 days/week), and drinking sweet beverages (i.e., lemonade, soda, carbonated soda, fruit juice, sugar sweetened dairy products, etc.) (recoded as ≤4 glasses/day, >4 glasses/day). The categories used for the behaviors are based on international recommendations [26, 27, 41, 42]. In this study, the four lifestyle-related behaviors of the child are hypothesized to be intermediate factors in the causal pathway between ethnic background and childhood overweight; therefore, we considered them to be potential mediators.

### 2.5. Statistical Analysis

Differences in baseline characteristics between the subgroups of non-Dutch children and Dutch children were examined using the Chi-square statistic for categorical variables and analysis of variance (ANOVA) for continuous variables. If the percentage of missing values in the study population did not exceed five percent, subjects with missing values on that variable were assigned to the most prevalent category for that variable [43]. If more than five percent were missing on a variable, a separate missing category was included for the analyses (this was only the case for the variables playing outside and watching TV). Multivariable logistic regression analyses were used to study the association between ethnic background of the child and being overweight (obesity included). Odds ratios (ORs) and 95% confidence intervals (CIs) were obtained for each ethnic subgroup and compared with the reference category (children of Dutch ethnic background).

The basic model investigated the association between ethnic background of the child and being overweight (obesity included). In the association with children's weight status, there were no interactions between sociodemographic characteristics, parental overweight, and ethnic background of the child (all *P* values > 0.10). Further, because the variables age and gender of the child and age of the parent appeared not to be confounders in the association between ethnic background of the child and being overweight, these variables were not included in the models.

First, the basic model was adjusted for the following confounders: gender of the parent, educational level of the parent, and family situation (model 1). In addition, we adjusted model 1 for the confounder parental overweight (model 2); the variable parental overweight (yes/no) was added as a separate step to the model, since it may reflect a genetic predisposition to overweight of the child and may reflect an environment that might be associated with behaviors predisposing to overweight in childhood [6, 7]. To test the influence of the potential mediating lifestyle-related behaviors of the child on the association between ethnic background and overweight (obesity included), the characteristics were added to model 2 one at a time. For each adjustment, the percentage change in OR was calculated for the subgroups of ethnic background ([OR_model  2  +  lifestyle-related  behavior_ − OR_model  2_]/[OR_model  2_ − 1] × 100) [44, 45]. A lifestyle-related behavior was considered relevant if the percentage change in the ORs for being overweight was >5% within an ethnic background subgroup. Subsequently, in model 3 (i.e., the final model), the association between ethnic background of the child and being overweight (obesity included) was adjusted also for the relevant mediating lifestyle-related behaviors; in this study, watching TV and having breakfast by the child adding the behaviors drinking sweet beverages and playing outside to the model resulted in changes in the ORs of <5%.

The analyses were performed using Statistical Package of Social Sciences version 17.0 for Windows.

## 3. Results

Table 1 shows the general characteristics of the total study population (*n* = 7801) and by ethnic background of the child. Mean age of the children was 5.7 (SD 0.4) years; 48.9% were girls. The prevalence of overweight (including obesity) was 8.2% among children of Dutch ethnic background, 19.1% among Moroccan, 27.4% among Turkish, 4.4% among Surinamese, and 17.2% among children of Dutch Antillean ethnic background (*P* < 0.01) (Table 1). There were statistically significant differences regarding the characteristics of the parent and family and regarding the lifestyle-related behaviors of the child between Dutch children and the subgroups of children with a non-Dutch ethnic background.

In the model with adjustment for the confounding characteristics (Table 2, model 1), the OR for being overweight (obesity included) among the Moroccan subgroup was 2.27 (95% CI 1.48–3.47), among Turkish children the OR was 3.63 (95% CI 2.46–5.35), among Dutch Antillean children the OR was 1.97 (95% CI 1.01–3.86), and the OR among Surinamese children was 0.47 (95% CI 0.20–1.06), compared to children with a Dutch ethnic background. After additional adjustment for parental overweight (Table 2, model 2), the ORs for being overweight for the non-Dutch ethnic subgroups decreased in the range from 7.5% to 27.8%.

In the final model (Table 3, model 3), with addition of the two relevant mediators (watching TV and having breakfast by the child), the ORs for being overweight among Moroccan children further decreased by 7.9% to 2.05 (95% CI 1.33–3.15), for Turkish children the OR further decreased by 12.2% to 3.02 (95% CI 2.02–4.50), and for Dutch Antillean children the OR further decreased by 8.6% to 1.64 (95% CI 0.83–3.25). For Surinamese children, the OR for being overweight in the final model was 0.41 (95% CI 0.18–0.95) (Table 3).

## 4. Discussion

This study shows that children with a Moroccan and Turkish ethnic background are at increased risk for being overweight (obesity included) compared to children of Dutch ethnic background. Adjustment for parental overweight decreased the odds for being overweight (obesity included) for children with a Moroccan ethnic background with 10.2% and for children with a Turkish ethnic background with 12.5%. Taking into account lifestyle-related behaviors of the child (watching TV and having breakfast) the odds further decreased by, respectively, 7.9% and 12.2%. The risk for being overweight among children of Dutch Antillean ethnic background did not differ significantly from Dutch children. Surinamese children had lower risk for being overweight.

For the present study a large sample (*n* = 7801) of young children throughout the Netherlands with a small age range was included; therefore, our results are specific to the 5-year-old age group. However, this was an opportunity sample of 9 out of 37 municipal health services that were able and willing to participate in the study. The prevalence of overweight (obesity included) in our study population was 8.2% for Dutch children, 19.1% for children of Moroccan ethnic background, and 27.4% for children of Turkish ethnic background. In comparison, the prevalence rates for 5 year olds presented by a nationwide study were approximately 15% for Dutch children, 26% for children of Moroccan ethnic background, and 31% for children of Turkish ethnic background [10]. Therefore, the prevalence of overweight (obesity included) is probably underestimated in our study, and results should be generalized with caution. However, clear increased risks for being overweight (obesity included) were found for children of Moroccan or Turkish ethnic background, and (although we cannot confirm this) we assume that our findings are the same in the source population of 5 year olds living in the Netherlands. 

There are also other methodological considerations that need to be addressed. Limitations of this study are the use of cross-sectional data and the use of self-reported data for the characteristics of the parents (including height and weight) and the children, which may have introduced bias such as recall bias. Further, parents may have given socially desirable answers, although anonymity was assured. It was not possible in the study to specifically assess energy intake/expenditure, for example, through a food frequency questionnaire and accelerometer. Further, in the present study no information was available on, for example, prenatal, perinatal, or postnatal factors (such as maternal smoking during pregnancy, birth weight, and receiving breastfeeding). Height and weight of the children were, however, measured by trained healthcare professionals of the municipal health services.

The term ethnic background is a social construct; it is constantly evolving, and it is not a fixed concept. Ethnic self-identification can change across generations or even change over time within a generation [17]. We based the definition of ethnic background of the child on country of birth, as defined by Statistics Netherlands [33], as this is the most objective and stable measure to use among young children in the context of the Netherlands [46]. With our data we could not evaluate indicators for the level of family acculturation in the society, which may be relevant for examining differences in the prevalence of overweight [47]. We therefore recommend future studies to also investigate a wide range of aspects related to ethnic background with regard to the association with childhood overweight, such as culture and ethnic identity [46].

It may be that ethnic background and indicators of socioeconomic status (SES), such as parental educational level, interact with each other in their association with childhood overweight [13, 17, 30, 48, 49]. This appeared not to be the case in our study; no interaction was found between parental educational level and ethnic background of the child in the association with children's weight status (see also the Methods section). This indicates that within the subgroup of children with a parent with a low educational level, children with a Moroccan or Turkish ethnic background are also at increased risk for being overweight, compared to Dutch children. This finding provides further evidence that the effect of ethnic background may be independent of the effect of SES on the risk for overweight among the children.

Our findings of a higher risk for being overweight among children with a Moroccan or Turkish ethnic background compared to children of Dutch ethnic background in the Netherlands are in line with the results of previous studies (which included study populations with other or wider age ranges) [10, 22, 23]. In contrast, it is known from the literature that in Turkey the prevalence of overweight among Turkish children aged 6–17 years is lower than in most European countries [50]. However, when people no longer live in their country of origin, their eating and drinking habits and physical activity behaviors may change [17]. 

Up to now, the prevalence of overweight among Surinamese and Dutch Antillean children was less well examined. One study found no differences in overweight prevalence between Dutch children and children of Surinamese South Asian ethnic background [22]. In our study, Surinamese children had a statistically significant lower risk for being overweight compared to Dutch children. It has been indicated in the literature that the average macronutrient intake of Surinamese children in the Netherlands is more in line with the guidelines for a healthy diet compared to the conventional Dutch diet [9]. So, Surinamese children might have healthier behaviors linked to diet. However, we were unable to confirm this with regard to the four lifestyle-related behaviors of the children in our study. On the contrary, we found that Surinamese children less often had breakfast daily and had a higher intake of sweet beverages compared to Dutch children. By interpreting the results for this subgroup, it should be taken into account that we included a relatively small group of children with a Surinamese ethnic background in our study population. Further, the composition of this subgroup might be mixed as Surinam is a multiethnic society with people originated from China, Indonesia, India, the Netherlands, and Africa [22]. So, future research should further investigate this potential lower risk for being overweight among a larger and varied group of Surinamese children, and in which more detailed information about diet and other lifestyle-related behaviors should be included. 

## 5. Conclusions

In conclusion, this study shows that already in 5-year-old children there are considerable differences in the prevalence of overweight (including obesity) between ethnic subgroups. Children of Moroccan and Turkish ethnic background are at increased risk for being overweight compared to children of Dutch ethnic background. Not all ethnic groups appeared to be at increased risk for being overweight; the prevalence of overweight among the subgroup with a Dutch Antilles ethnic background did not differ significantly from Dutch children, and the prevalence among Surinamese children was lower. The higher risk for Moroccan and Turkish children is explained by parental weight status for >10%. Also, the behaviors watching TV and not having breakfast by the child appeared to contribute in explaining the higher risk for Moroccan and Turkish children (resp., for 7.9% and 12.2%). We recommend that future studies investigate parenting factors, social-cultural determinants, prenatal, perinatal, and postnatal factors, and specific measures of diet, sedentary, and physical activity behaviors over time, to further explain differences in prevalence of early childhood overweight among ethnic subgroups living in the same country. When developing overweight prevention programs for young children, for example, for use during well-child or pediatric visits to counsel and advise parents, attention should be paid to the differences in risk across ethnic subgroups. As parental weight status and the lifestyle-related behaviors watching TV and having breakfast by the child appear to contribute to the increased risk for Turkish and Moroccan subgroups, these factors should be taken into account by tailoring the interventions to the specific subgroups involved. 

## Figures and Tables

**Table 1:** General characteristics of the total study population and by ethnic background of the child (*n* = 7801).

	Total (*n* = 7801)	Dutch (*n* = 7302) (reference group)	Moroccan (*n* = 152)	Turkish (*n* = 146)	Surinamese (*n* = 137)	Dutch Antillean (*n* = 64)
Child characteristics						
Gender of child						
Girl (%)	48.9	48.9	49.3	50.7	46.0	50.0
Age of child (years)						
Mean (SD)	5.7 (0.4)	5.7 (0.4)	5.8 (0.5)	5.8 (0.5)	5.9 (0.4)	5.7 (0.5)
Overweight child						
Overweight (obesity included) (%)	8.8	8.2	19.1**	27.4**	4.4	17.2**
Characteristics of parent						
Gender of responding parent						
Woman (%)	89.9	90.9	67.8**	65.1**	83.9**	93.8
Age of parent (years)						
Mean (SD)	36.6 (4.5)	36.7 (4.4)	36.1 (6.9)	33.8 (4.8)**	36.7 (5.6)	35.6 (5.9)
Educational level of parent						
Low (%)	21.8	20.6	47.7	54.6	23.5	22.6
Mid (%)	45.3	45.4	41.7	36.9	50.7	58.4
High (%)	32.8	33.9	10.6**	8.5**	25.7	29.0
Parental overweight						
Overweight (obesity included) (%)^a^	31.1	29.9	48.0**	56.8**	40.1	48.4**
Family characteristics						
Family situation						
Two-parent family (%)	93.3	94.1	87.5	91.8	78.1	59.4
Otherwise (%)	6.7	5.9	12.5**	8.2	21.9**	40.6^∗∗b^
Lifestyle-related behaviors child						
Playing outside						
≥1 hour/day (%)	83.3	83.8	72.4	76.7	79.6	75.0
<1 hour/day (%)	5.4	5.4	6.6	4.8	9.5	1.6
Missing (%)	11.3	10.8	21.1**	18.5*	10.9	23.4^∗∗b^
Watching TV						
≤2 hours/day (%)	76.7	78.6	39.5	45.9	61.3	54.7
>2 hours/day (%)	16.2	14.9	42.8	37.7	28.5	28.1
Missing (%)	7.1	6.6	17.8**	16.4**	10.2**	17.2^∗∗b^
Having breakfast						
7 days/week (%)	93.5	94.4	87.5	65.1	86.9	87.5
<7 days/week (%)	6.5	5.6	12.5**	34.9**	13.1**	12.5^∗b^
Sweet beverages						
≤4/day (%)	84.0	84.4	77.6	84.9	74.5	70.3
>4 day (%)	16.0	15.6	22.4*	15.1	25.5**	29.7**

SD: standard deviation.

*P* values are for chi-squared tests (categorical factors) or one-way analysis of variance (continuous factors).

*P < 0.05, **P < 0.01.

^a^Overweight (obesity included) = BMI ≥ 25 (kg/m^2^) [3].

^b^ *P* value also based on Fisher's exact test because of small groups.

**Table 2:** Logistic regression analyses for the association between ethnic background and overweight (obesity included) among 5 year olds and change in ORs after adjustment for lifestyle-related behaviors of the child (*n* = 7801).

	Dutch (ref) OR	Moroccan OR (95% CI)	Change *a* ^a^ (%)	Turkish OR (95% CI)	Change *b* ^a^ (%)	Surinamese OR (95% CI)	Change *c* ^a^ (%)	Dutch Antillean OR (95% CI)	Change *d* ^a^ (%)
Model 1	**1.00**	**2.27 (1.48–3.47)**		**3.63 (2.46–5.35)**		0.47 (0.20–1.06)		**1.97 (1.01–3.86)**	
Model 2	**1.00**	**2.14 (1.39–3.28)**	−10.2	**3.30 (2.23–4.87)**	−12.5	**0.43 (0.19–0.98)**	−7.5	1.70 (0.86–3.35)	−27.8

Lifestyle-related behaviors child			Change *a* ^b^ (%)		Change *b* ^b^ (%)		Change *c* ^b^ (%)		Change *d* ^b^ (%)

Model 2 + playing outside	**1.00**	**2.14 (1.40–3.29)**	0.0	**3.30 (2.24–4.88)**	0.0	**0.43 (0.19–0.98)**	0.0	1.71 (0.87–3.37)	−1.4
Model 2 + watching TV	**1.00**	**2.05 (1.33–3.16)**	−7.9	**3.21 (2.16–4.75)**	−3.9	**0.42 (0.18–0.96)**	+1.8	1.67 (0.84–3.29)	−4.3
Model 2 + having breakfast	**1.00**	**2.12 (1.38–3.26)**	−1.8	**3.09 (2.07–4.60)**	−9.1	**0.42 (0.18–0.96)**	+1.8	1.68 (0.85–3.31)	−2.9
Model 2 + sweet beverages	**1.00**	**2.13 (1.39–3.27)**	−0.9	**3.32 (2.24–4.90)**	+0.9	**0.42 (0.18–0.97)**	+1.8	1.68 (0.85–3.32)	−2.9

OR: odds ratio, CI: confidence interval.

Model 1: ethnic background of the child + gender of the parent, educational level of the parent, and family situation.

Model 2: model 1 + parental overweight (overweight (obesity included) = BMI ≥ 25 (kg/m^2^)) [3].

^a^Change *a*, *b*, *c* and change *d* represent the respective changes in OR for children with a Moroccan, Turkish, Surinamese, and Dutch Antillean ethnic background relative to model 1, after adjustment for parental overweight ([OR_model 2_ − OR_model 1_]/[OR_model 1_ − 1] × 100).

^b^Change *a*, *b*, *c* and change *d* represent the respective changes in OR for children with a Moroccan, Turkish, Surinamese, and Dutch Antillean ethnic background relative to model 2, after individual adjustment for lifestyle-related behaviors of the child ([OR_model 2 + lifestyle-related behavior_ − OR_model 2_]/[OR_model 2_ −  1] × 100).

**Table 3:** Logistic regression analyses for association between ethnic background and overweight (obesity included) among 5 year olds, after adjustment for confounders and mediators (*n* = 7801).

Ethnic background child	Model 2 OR (95% CI)	Model 3 OR (95% CI)	Change *a* ^a^ (%)
Dutch	**1.00 (reference)**	**1.00 (reference)**	
Moroccan	**2.14 (1.39–3.28)**	**2.05 (1.33–3.15)**	−7.9%
Turkish	**3.30 (2.23–4.87)**	**3.02 (2.02–4.50)**	−12.2%
Surinamese	**0.43 (0.19–0.98)**	**0.41 (0.18–0.95)**	+3.5%
Dutch Antillean	1.70 (0.86–3.35)	1.64 (0.83–3.25)	−8.6%

OR: odds ratio; CI: confidence interval.

Model 2: ethnic background of the child + gender of the parent, educational level of the parent, and family situation + parental overweight (overweight (obesity included) = BMI ≥ 25 (kg/m^2^)) [3].

Model 3: model 2 + relevant lifestyle-related behaviors of the child (watching TV, having breakfast) (see Table 2).

^a^Change *a* represents the respective change in OR for children with a Moroccan, Turkish, Surinamese, and Dutch Antillean ethnic background relative to model 2, after adjustment for mediators, the lifestyle-related behaviors of the child (model 3) ([OR_model 3_ − OR_model 2_]/[OR_model 2_ − 1] × 100).

## References

[1] Ebbeling CB, Pawlak DB, Ludwig DS (2002). Childhood obesity: public-health crisis, common sense cure. *Lancet*.

[2] Lobstein T, Baur L, Uauy R (2004). Obesity in children and young people: a crisis in public health. *Obesity Reviews*.

[3] World Health Organization Obesity and overweight. *Factsheet*.

[4] Dietz WH (1998). Health consequences of obesity in youth: childhood predictors of adult disease. *Pediatrics*.

[5] Rennie KL, Johnson L, Jebb SA (2005). Behavioural determinants of obesity. *Best Practice and Research*.

[6] Kumanyika SK (2008). Environmental influences on childhood obesity: ethnic and cultural influences in context. *Physiology and Behavior*.

[7] Ritchie LD, Welk G, Styne D, Gerstein DE, Crawford PB (2005). Family environment and pediatric overweight: what is a parent to do?. *Journal of the American Dietetic Association*.

[8] Danielzik S, Czerwinski-Mast M, Langnäse K, Dilba B, Müller MJ (2004). Parental overweight, socioeconomic status and high birth weight are the major determinants of overweight and obesity in 5-7 y-old children: baseline data of the Kiel Obesity Prevention Study (KOPS). *International Journal of Obesity*.

[9] Brussaard JH, van Erp-Baart MA, Brants HAM, Hulshof KFAM, Löwik MRH (2001). Nutrition and health among migrants in the Netherlands. *Public Health Nutrition*.

[10] Fredriks AM, van Buuren S, Hira Sing RA, Wit JM, Verloove-Vanhorick SP (2005). Alarming prevalences of overweight and obesity for children of Turkish, Moroccan and Dutch origin in the Netherlands according to international standards. *Acta Paediatrica*.

[11] Wang Y, Beydoun MA (2007). The obesity epidemic in the United States—gender, age, socioeconomic, racial/ethnic, and geographic characteristics: a systematic review and meta-regression analysis. *Epidemiologic Reviews*.

[12] Kuepper-Nybelen J, Lamerz A, Bruning N, Hebebrand J, Herpertz-Dahlmann B, Brenner H (2005). Major differences in prevalence of overweight according to nationality in preschool children living in Germany: determinants and public health implications. *Archives of Disease in Childhood*.

[13] Singh GK, Kogan MD, van Dyck PC, Siahpush M (2008). Racial/ethnic, socioeconomic, and behavioral determinants of childhood and adolescent obesity in the United States: analyzing independent and joint associations. *Annals of Epidemiology*.

[14] Kirchengast S, Schober E (2006). To be an immigrant: a risk factor for developing overweight and obesity during childhood and adolescence?. *Journal of Biosocial Science*.

[15] Bongard S, Pogge SF, Arslaner H, Rohrmann S, Hodapp V (2002). Acculturation and cardiovascular reactivity of second-generation Turkish migrants in Germany. *Journal of Psychosomatic Research*.

[16] Kimbro RT, Brooks-Gunn J, McLanahan S (2007). Racial and ethnic differentials in overweight and obesity among 3-year-old children. *American Journal of Public Health*.

[17] Caprio S, Daniels SR, Drewnowski A (2008). Influence of race, ethnicity, and culture on childhood obesity: implications for prevention and treatment. *Obesity*.

[18] Taveras EM, Gillman MW, Kleinman K, Rich-Edwards JW, Rifas-Shiman SL (2010). Racial/ethnic differences in early-life risk factors for childhood obesity. *Pediatrics*.

[19] Wang Y (2011). Disparities in pediatric obesity in the United States. *Advances in Nutrition*.

[20] Rossen LM, Schoendorf KC (2012). Measuring health disparities: trends in racial-ethnic and socioeconomic disparities in obesity among 2- to 18-year old youth in the United States, 2001–2010. *Annals of Epidemiology*.

[21] El-Sayed AM, Scarborough P, Galea S (2011). Ethnic inequalities in obesity among children and adults in the UK: a systematic review of the literature. *Obesity Reviews*.

[22] de Wilde JA, van Dommelen P, Middelkoop BJC, Verkerk PH (2009). Trends in overweight and obesity prevalence in Dutch, Turkish, Moroccan and Surinamese South Asian children in the Netherlands. *Archives of Disease in Childhood*.

[23] de Hoog ML, van Eijsden M, Stronks K, Gemke RJ, Vrijkotte TG (2011). Overweight at age two years in a multi-ethnic cohort (ABCD study): the role of prenatal factors, birth outcomes and postnatal factors. *BMC Public Health*.

[24] Whitaker RC (2003). Obesity prevention in pediatric primary care: four behaviors to target. *Archives of Pediatrics and Adolescent Medicine*.

[25] Bulk-Bunschoten AMW, Renders CM, van Leerdam FJM, HiraSing RA (2005). *Youth Health Care Overweight-Prevention-Protocol*.

[26] Davis MM, Gance-Cleveland B, Hassink S, Johnson R, Paradis G, Resnicow K (2007). Recommendations for prevention of childhood obesity. *Pediatrics*.

[27] Barlow SE (2007). Expert committee recommendations regarding the prevention, assessment, and treatment of child and adolescent overweight and obesity: summary report. *Pediatrics*.

[28] Moreno LA, Rodriguez G (2007). Dietary risk factors for development of childhood obesity. *Current Opinion in Clinical Nutrition & Metabolic Care*.

[29] Harding S, Teyhan A, Maynard MJ, Cruickshank JK (2008). Ethnic differences in overweight and obesity in early adolescence in the MRC DASH study: the role of adolescent and parental lifestyle. *International Journal of Epidemiology*.

[30] Waters E, Ashbolt R, Gibbs L (2008). Double disadvantage: the influence of ethnicity over socioeconomic position on childhood overweight and obesity: findings from an inner urban population of primary school children. *International Journal of Pediatric Obesity*.

[31] Veldhuis L, Struijk MK, Kroeze W (2009). “Be active, eat right”, evaluation of an overweight prevention protocol among 5-year-old children: design of a cluster randomised controlled trial. *BMC Public Health*.

[32] Renders CM, Halberstadt J, Frenkel CS, Rosenmöller P, Seidell JC, Hirasing RA (2010). Tackling the problem of overweight and obesity: the Dutch approach. *Obesity Facts*.

[33] Centraal Bureau voor de Statistiek (Statistics Netherlands) (2004). *Migrants in the Netherlands 2004*.

[34] Bulk-Bunschoten AMW, Renders CM, van Leerdam FJM, HiraSing RA (2004). *Youth Health Care Overweight-Detection-Protocol*.

[35] Cole TJ, Bellizzi MC, Flegal KM, Dietz WH (2000). Establishing a standard definition for child overweight and obesity worldwide: international survey. *British Medical Journal*.

[36] Malik VS, Schulze MB, Hu FB (2006). Intake of sugar-sweetened beverages and weight gain: a systematic review. *American Journal of Clinical Nutrition*.

[37] Anderson SE, Economos CD, Must A (2008). Active play and screen time in US children aged 4 to 11 years in relation to sociodemographic and weight status characteristics: a nationally representative cross-sectional analysis. *BMC Public Health*.

[38] Laurson KR, Eisenmann JC, Welk GJ, Wickel EE, Gentile DA, Walsh DA (2008). Combined influence of physical activity and screen time recommendations on childhood overweight. *Journal of Pediatrics*.

[39] Rampersaud GC, Pereira MA, Girard BL, Adams J, Metzl JD (2005). Breakfast habits, nutritional status, body weight, and academic performance in children and adolescents. *Journal of the American Dietetic Association*.

[40] Centraal Bureau voor de Statistiek (Statistics Netherlands) (2004). *The Dutch Standard Classification of Education*.

[41] American Academy of Pediatrics (2001). Committee on public education: children, adolescents, and television. *Pediatrics*.

[42] American Academy of Pediatrics (2001). Committee on nutrition: the use and misuse of fruit juice in pediatrics. *Pediatrics*.

[43] Harrell FE (2001). *Regression Modeling Strategies: With Applications to Linear Models, Logistic Regression, and Survival Analysis*.

[44] van de Mheen H, Stronks K, van den Bos J, Mackenbach JP (1997). The contribution of childhood environment to the explanation of socio-economic inequalities in health in adult life: a retrospective study. *Social Science and Medicine*.

[45] Silva LM, Coolman M, Steegers EAP (2008). Low socioeconomic status is a risk factor for preeclampsia: the generation R study. *Journal of Hypertension*.

[46] Stronks K, Kulu-Glasgow I, Agyemang C (2009). The utility of “country of birth” for the classification of ethnic groups in health research: the Dutch experience. *Ethnicity & Health*.

[47] Sussner KM, Lindsay AC, Peterson KE (2009). The influence of maternal acculturation on child body mass index at age 24 months. *Journal of the American Dietetic Association*.

[48] Braveman PA, Cubbin C, Egerter S (2005). Socioeconomic status in health research: one size does not fit all. *Journal of the American Medical Association*.

[49] Sue S, Dhindsa MK (2006). Ethnic and racial health disparities research: issues and problems. *Health Education and Behavior*.

[50] Simsek E, Akpinar S, Bahcebasi T, Senses DA, Kocabay K (2008). The prevalence of overweight and obese children aged 6–17 years in the West Black Sea region of Turkey. *International Journal of Clinical Practice*.

